# Pathophysiology Annual Report Card 2025

**DOI:** 10.3390/pathophysiology33010017

**Published:** 2026-02-13

**Authors:** Jonathan Steven Alexander

**Affiliations:** Department of Molecular and Cellular Physiology, LSU Health Shreveport, Shreveport, LA 71103, USA; jonathan.alexander@lsuhs.edu

As the Year of the Snake draws to a close and we ‘gallop’ into the Year of the Horse, we find a fitting metaphor for the work of science: continued effort, coordination, and progress that depends as much on balance in the saddle as on speed. This moment provides a moment to pause and an opportunity to reflect on 2025 at *Pathophysiology*, and to once more think about how our journal and the field it represents, Pathophysiology, continue to evolve.

Everyone at MDPI working on the journal and all of the schools and hospitals participating with us by publishing through *Pathophysiology* in 2025 have communicated that they are extremely pleased with the increasing readership and exposure. The number of submissions to the journal has continued to increase from all over the world, with work evaluating pathomolecular mechanisms and the improved integration of pathophysiology into translational research. The vast expanse of topics covered by the journal illustrates a growing recognition that *Pathophysiology* is seated at the intersection of pathomechanism discovery and the clinical application of these findings. Equally important is the fact that the studies and reviews published in our journal are being widely consumed—another reminder that scientific communication only really fulfills its purpose when it reaches its target audience.

This progress in 2025 reflects the considerable collective efforts of so many individuals, especially our extraordinary editorial teams in China, Serbia, and Switzerland. We would like to extend our sincere thanks to them all for making the review and publication processes at *Pathophysiology* rapid, fair, and transparent. At the same time, we also say a huge thank you to all of the authors who entrust us with their carefully produced manuscripts and reviews, and, of course, to our reviewers for their thoughtful (and usually unseen) efforts. Peer review remains a demanding role and is one of the most generous gifts you can give as academic; the value to the process cannot be overstated. We very deeply appreciate your contributions here. To our Editorial Board Members, Section Editors, and managing staff—our journal could not exist without you and your hard work, allowing our journal to operate efficiently. Thank you sincerely.

*Pathophysiology* continues to remain committed to Open Access publishing. Research in the biomedical sciences is most frequently supported by public funding, and therefore we have a responsibility to ensure that the outcomes of these studies are made accessible to clinicians, researchers, and the broader public who support this work. Open dissemination of the research also promotes transparency, encourages dialogue, and informs evidence-based clinical practice and policy. In an effort to accomplish this, we often guide authors to adjust their report titles as statements of findings.

Another notable development across the health sciences is the increasing emphasis on showing the relevance of submitted findings beyond academics or the laboratory. While pathophysiological research often starts with mechanistic insights leading to experimental models, there is now a widening appreciation for connecting such findings to clinical scenarios where diagnostics and treatments can be improved through these efforts. While engagement with clinicians, patients, and other stakeholders strengthens, the interpretation of this work ensures that discoveries make the entire voyage from bench to bedside. Thus, *Pathophysiology* is and intends to remain an important guide for clinicians as well as academics.

*Pathophysiology* has also observed a continued rise in the submission of reviews of existing literature. Such reviews perform an essential function in explaining evidence and identifying where gaps in knowledge really exist. At the same time, not every area of the literature requires such consideration. As an editorial policy, *Pathophysiology* continues to encourage well-conceived reviews while also carefully determining whether they accurately and fairly ‘synthesize’ the most unanimous consensus of the topic’s scope, methodology, and contributions to the field.

In 2025, *Pathophysiology* published numerous widely read studies that increased our appreciation of metabolic, infectious, and inflammatory disease mechanisms. Several of these were (to me) particularly interesting, especially the study by Nhau et al. [1] demonstrating that SARS-CoV-2’s main protease can directly dysregulate liver insulin and glucose networks, which supports mechanistic links to long and post-COVID diabetogenesis and pathophysiology. Additionally, another valuable insight into possible long COVID pathophysiology by Kuliyeva et al. [2] described mast cell clustering, fibrosis, and vascular thrombosis in post-COVID femoral head osteonecrosis, triggering immune and vascular effects possibly explaining post-COVID osteoarthritis and changes in bone health. While all of the work published in 2025 is highly valuable, these reports may help to explain molecular mechanisms in clinical pathologies across infectious, metabolic, and inflammatory diseases.

Several administrative improvements were also made in 2025. Many new topic sections were introduced under the supervision of the In-Field of Expertise (IFE) Section Editors-in-Chief. These changes are intended to highlight the most important and emerging areas in Pathophysiology and create highly focused scholarly research communities and clinicians with a high level of familiarity and influence in these fields.

Throughout 2025, *Pathophysiology* also engaged actively with the wider research community through participation in conferences and the development of reviewer activities, and by recognizing outstanding scholarly contributions. These efforts confirm our belief that journals do not merely ‘archive’ research, but actively contribute to the ongoing development of the disciplines they serve. In addition, and very importantly, *Pathophysiology* has also continued to invest in academic excellence by offering substantial awards to a number of early-stage scientists and supporting several scientific meetings.

We at *Pathophysiology* are happy to receive news of any upcoming conferences or meetings that may be of interest to the readers of the journal in 2026, and we may be able to consider helping to support some of them. These efforts supported by MDPI and *Pathophysiology* can improve support to our community and help to mentor a next generation of readers, editors, and reviewers—this is an important investment in the future of scientific publishing.

Looking ahead in 2026, we, your *Pathophysiology* editorial team, are deeply committed to publishing high impact, rigorous, meaningful, and constructive peer-reviewed research and to provide our authors with a transparent and respectful process for all contributors. The ‘Year of the Horse’ may suggest that meaningful progress is not a short ‘sprint’ but is accomplished through gained momentum and careful steering. We are eager to continue this journey together in the year(s) to come!

## References

[1] Nhau P.T., Gamede M., Khathi A., Sibiya N. (2025). SARS-CoV-2 Main Protease Dysregulates Hepatic Insulin Signaling and Glucose Uptake: Implications for Post-COVID-19 Diabetogenesis. Pathophysiology.

[2] Kuliyeva A., Serejnikova N., Eshmotova G., Teslya Y., Ivina A., Zarov A., Panin M., Prizov A., Lyalina V., Shestakov D. (2025). Post-COVID-19 Femoral Head Osteonecrosis Exhibits Mast Cell Clusters, Fibrosis, and Vascular Thrombosis: Key Pathological Mechanisms in Long COVID-19 Bone Degeneration. Pathophysiology.

